# Comparing Poor and Favorable Outcome Prediction With Machine Learning After Mechanical Thrombectomy in Acute Ischemic Stroke

**DOI:** 10.3389/fneur.2022.737667

**Published:** 2022-05-27

**Authors:** Matthias A. Mutke, Vince I. Madai, Adam Hilbert, Esra Zihni, Arne Potreck, Charlotte S. Weyland, Markus A. Möhlenbruch, Sabine Heiland, Peter A. Ringleb, Simon Nagel, Martin Bendszus, Dietmar Frey

**Affiliations:** ^1^Department of Neuroradiology, Heidelberg University Hospital, Heidelberg, Germany; ^2^Charité Lab for Artificial Intelligence in Medicine, Charité Universitätsmedizin Berlin, Berlin, Germany; ^3^QUEST (Quality, Ethics, Open Science, Translation) Center for Responsible Research at Berlin Institute of Health, Charité Universitätsmedizin Berlin, Berlin, Germany; ^4^School of Computing and Digital Technology, Faculty of Computing, Engineering and the Built Environment, Birmingham City University, Birmingham, United Kingdom; ^5^School of Computing, Technological University Dublin, Dublin, Ireland; ^6^Department of Neurology, Heidelberg University Hospital, Heidelberg, Germany

**Keywords:** stroke, mechanical thrombectomy, outcome prediction, machine learning, MRI, perfusion imaging, mismatch

## Abstract

**Background and Purpose:**

Outcome prediction after mechanical thrombectomy (MT) in patients with acute ischemic stroke (AIS) and large vessel occlusion (LVO) is commonly performed by focusing on favorable outcome (modified Rankin Scale, mRS 0–2) after 3 months but poor outcome representing severe disability and mortality (mRS 5 and 6) might be of equal importance for clinical decision-making.

**Methods:**

We retrospectively analyzed patients with AIS and LVO undergoing MT from 2009 to 2018. Prognostic variables were grouped in baseline clinical (A), MRI-derived variables including mismatch [apparent diffusion coefficient (ADC) and time-to-maximum (Tmax) lesion volume] (B), and variables reflecting speed and extent of reperfusion (C) [modified treatment in cerebral ischemia (mTICI) score and time from onset to mTICI]. Three different scenarios were analyzed: (1) baseline clinical parameters only, (2) baseline clinical and MRI-derived parameters, and (3) all baseline clinical, imaging-derived, and reperfusion-associated parameters. For each scenario, we assessed prediction for favorable and poor outcome with seven different machine learning algorithms.

**Results:**

In 210 patients, prediction of favorable outcome was improved after including speed and extent of recanalization [highest area under the curve (AUC) 0.73] compared to using baseline clinical variables only (highest AUC 0.67). Prediction of poor outcome remained stable by using baseline clinical variables only (highest AUC 0.71) and did not improve further by additional variables. Prediction of favorable and poor outcomes was not improved by adding MR-mismatch variables. Most important baseline clinical variables for both outcomes were age, National Institutes of Health Stroke Scale, and premorbid mRS.

**Conclusions:**

Our results suggest that a prediction of poor outcome after AIS and MT could be made based on clinical baseline variables only. Speed and extent of MT did improve prediction for a favorable outcome but is not relevant for poor outcome. An MR mismatch with small ischemic core and larger penumbral tissue showed no predictive importance.

## Introduction

Mechanical thrombectomy (MT) is the most effective treatment for patients with acute ischemic stroke (AIS) due to a large vessel occlusion of the anterior circulation (1). While the average treatment effect and outcome benefit across the entire group of patients is large, outcome still differs significantly for individual patients (1). Multiple prognostic variables and their combination render individual outcome prognosis after MT difficult. For example, in a group of patients with successful and fast reperfusion, ~60% still had an unfavorable outcome (mRS 3–6 after 3 months) (2). At present, the relative importance and combination of single prognostic variables for individual outcome prediction is still a matter of debate.

One way to address this problem is to utilize artificial intelligence, in particular machine learning (ML) approaches. These models are potentially superior to conventional linear or logistic regression models as they excel at finding complex and non-linear relationships across a multitude of prognostic variables. Specially, artificial neural networks and methods of tree-boosting are promising tools in this regard (3). Recent advances have made it possible to uncover which individual prognostic variables are most important in such models (4, 5) based on a feature importance analysis.

Applying this methodology to outcome prediction after MT, multiple prognostic variables can be used representing the clinical course of stroke patients: baseline clinical variables (1), MRI variables including perfusion and infarct core (6), and finally, variables assessing the speed and extent of reperfusion (7).

The outcome and potential benefit of MT are usually assessed after 3 months with the modified Rankin Scale (mRS) in a dichotomized analysis: 0–2 is defined as favorable outcome and the remaining 3–6 as unfavorable outcome. Patients with a predicted favorable outcome will undoubtedly undergo MT. However, the remaining group of patients with unfavorable outcome is highly heterogeneous, ranging from outcomes of mRS of 3 (moderate disability) to 6 (death).

Therefore, it may also be reasonable to find prognostic factors for a poor outcome (8) (severe disability or death after 3 months, with an mRS score of 5 or 6). In those patients, withholding treatment could be discussed.

Therefore, in the presented work we used ML to predict outcome after MT directly comparing two different dichotomization paradigms: for favorable (mRS 0–2 vs. 3–6) and poor outcome (mRS 5 and 6 vs. 1–4) with multiple prognostic variables grouped in three sets: baseline clinical, MRI–derived, and MT-associated variables.

## Methods

The data that support the findings of this study are available from the corresponding author upon reasonable request.

The study protocol for this retrospective analysis of our prospectively established stroke database was approved by the ethics committee of Heidelberg University and patient-informed consent was waived.

### Patients

We identified patients with AIS due to an occlusion of the middle cerebral artery in the M1 or M2 segment or the distal terminus of the internal carotid artery who were treated with MT between 03/2009 and 09/2018; 95/210 patients were treated between 2010 and 2013, and the remaining 116/210 between 2014 and 2018. Between these two groups, there was no significant outcome difference after 3 months (Mann–Whitney test, *p* = 0.83).

Patients were treated at a single center (University Hospital Heidelberg). The attending neurologist and interventional neuroradiologist decided on treatment on a case-by-case basis. Only patients with a completed MRI protocol and outcome assessment at 3 months were included.

Baseline clinical and imaging parameters are given in Table 1. Individual patient outcome was the score on the mRS (9) at 3 months assessed by a standardized interview (unblinded investigator per phone call or a personal letter to the patient). The mTICI was used to grade recanalization on final angiographic images (10). A score of mTICI 2b or better on final angiogram was regarded as successful reperfusion.

**Table 1:** Prognostic variables (features).

		**All patients** **(*n* = 210)**	**Favorable outcome** **(*n* = 83/210)**	**Poor outcome** **(*n* = 49/210)**
**Set A: Baseline clinical variables**				
Time from stroke onset to MR-imaging (time in minutes)	Median/IQR	260 (126–569)	257 (123–552)	219 (109–526)
Wake up stroke	%	85 (40%)	31 (37%)	21 (43%)
Age (years)	Median/IQR	72 (59–78)	69 (53–75)	76 (68–81)
Sex	Male/female	92/118	39/44	21/28
Diabetes	%	37 (18%)	7 (8%)	14 (29%)
Hypertonia	%	131 (62%)	47 (57%)	37 (76%)
Coronary heart disease	%	36 (17%)	11 (13%)	12 (24%)
Arrhythmia/atrial fibrillation	%	77 (37%)	22 (27%)	24 (49%)
Hyperlipidemia	%	62 (30%)	23 (28%)	16 (33%)
NIHSS scale at admission (0–42)	Median/IQR	16 (12–20)	15 (10–20)	20 (15–30)
mRS pre-stroke (0–5)	Median/IQR	0 (0–1)	0 (0–1)	1 (0–2)
i.v. Thrombolysis	%	132 (63%)	58 (70%)	29 (59%)
**Set B: Magnetic resonance imaging derived variables**				
ADC lesion volume (ml)	Median/IQR	14 (8–30)	16 (7–32)	14 (9–27)
Tmax lesion volume (ml)	Median/IQR	78 (39–140)	73 (36–121)	105 (60–168)
Mismatch ratio (Tmax lesion volume/ADC lesion volume)	Median/IQR	4.6 (2.3–8.4)	4.2 (2.1–8.4)	5.3 (2.9–13.3)
Occlusion distal carotid artery	%	12 (6%)	7 (8%)	2 (4%)
Occlusion carotid terminus	%	33 (16%)	14 (17%)	7 (14%)
Occlusion M1 segment middle cerebral artery	%	131 (62%)	49 (59%)	32 (65%)
Occlusion M2 segment middle cerebral artery	%	36 (17%)	14 (17%)	7 (14%)
**Set C: Thrombectomy associated variables**				
Final mTICI score (TICI 3 and 2b)	%	154 (73%)	73 (88%)	26 (53%)
Time from stroke onset to final mTICI score	Median/IQR	492 (330–787)	526 (319–853)	434 (319–721)

*Prognostic variables were grouped in three distinct sets: Baseline clinical variables (A), MRI-derived mismatch variables (B), and mechanical thrombectomy-associated variables (C). Variables are given for all patients and the two subgroups of patients with favorable outcome (mRS 0–2) and with poor outcome (mRS 5 and 6) after 3 months*.

### MRI Protocol

In a routine clinical setting, MR images were acquired on 3 Tesla MRI systems (Magnetom Verio, TIM Trio and Magnetom Prisma; Siemens Healthcare, Erlangen, Germany). Imaging protocol included diffusion-weighted, FLAIR, susceptibility weighted and T2-weighted sequences, non-contrast time-of-flight, and contrast-enhanced angiography as well as dynamic susceptibility contrast perfusion-weighted imaging. The imaging protocol has been published previously and is included in the Supplementary Material (11).

### Image Post-processing

All image analysis was performed blinded to clinical outcome. Diffusion-weighted imaging (DWI) and perfusion MRI images were post-processed with Olea Sphere^®^ (Olea Medical^®^, La Ciotat, France). ADC maps were automatically calculated from DWI images with different *b*-values. For perfusion imaging, automatic motion correction was applied. The arterial input function (AIF) was detected automatically. In two cases, the automatically detected AIF was manually corrected. Tmax maps were calculated using a block-circulant singular-value decomposition (cSVD) deconvolution algorithm. Diffusion lesion volumes [ADC value threshold of ≤620 × 10^−6^ mm^2^/s (12)] and Tmax lesion volumes [Tmax threshold ≥ 6 s (13)] were segmented semiautomatically and manually corrected for artifacts by a neuroradiologist (MM) with more than 6 years of experience in stroke imaging.

### ML Framework

For the training of the ML models, we utilized a publicly available ML framework for predictive modeling. The program code is available on Github (https://github.com/prediction2020/explainable-predictive-models). Details on the technical implementation have been described in open-access publications previously (5).

### Definition of Prognostic Paradigms

We defined two distinct prognostic paradigms: For the first paradigm **I**, all patients included in the study were dichotomized in favorable outcome with an mRS of 0, 1, or 2 at 3 months vs. the remaining with mRS 3–6. For the second paradigm **II**, again all patients were included and dichotomized but in poor outcome, defined as mRS 5 or 6 at 3 months vs. the remaining with mRS 0–4. The dichotomized mRS was used as a label for the ML analysis.

### Prognostic Variables for Input Feature Definition

We grouped prognostic variables in three distinct sets (Table 1): Baseline clinical variables (A), MRI-derived variables (B), and thrombectomy-related variables (C). The mTICI score as a measure for the success of reperfusion was dichotomized for the final analysis (mTICI 2b/3 vs. 0–2a). The prognostic variables included in the three sets were used as input features for the ML analysis. Mismatch ratio was not included as an independent feature because it is derived from the ADC and TMax lesion volumes and would be redundant information. Target mismatch was defined according to the EXTEND-IA study (6) with an infarct core of <70 ml on ADC maps, a ratio of Tmax Lesion volume to ADC lesion volume of 1.2 or higher and an absolute mismatch volume of 10 ml or more.

We defined three distinct prediction scenarios with the different sets of prognostic variables: Prediction with baseline clinical variables only (A), with baseline clinical and MRI variables combined (A+B), and finally with all baseline clinical, MRI and thrombectomy-associated variables combined (A+B+C).

**Figure 1:**
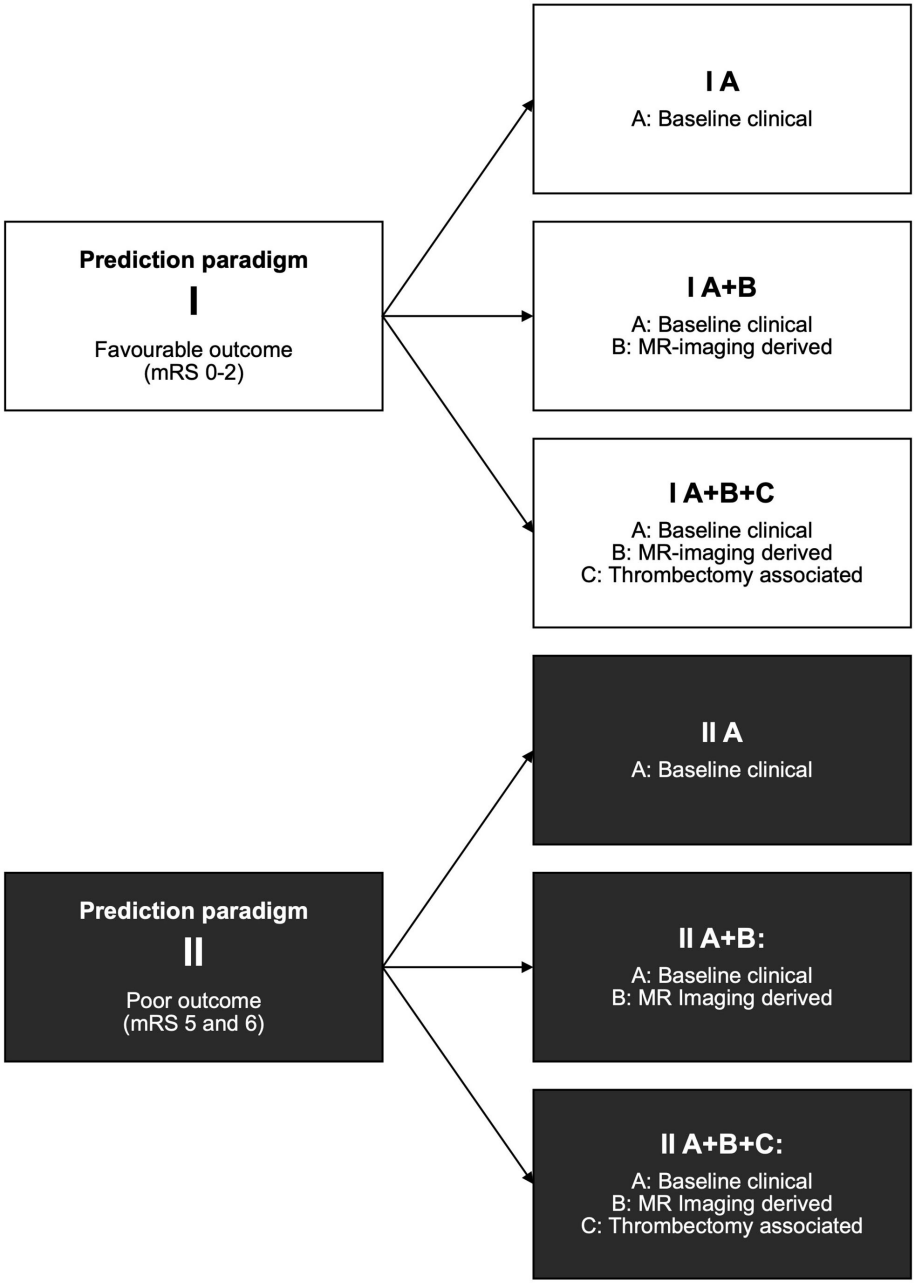
Prediction paradigms and resulting scenarios. For each paradigm, all patients included in the study were dichotomized: Paradigm I for favorable outcome with mRS 0–2 at 3 months (vs. the remaining 3–6) and paradigm II for poor outcome with mRS 5 and 6 (vs. the remaining 0–4). For the prediction scenarios, three sets of prediction variables A, B, and C were consecutively added. For an overview of prediction variables included in the sets, see Table 1. The combination of each of the three prediction variable sets and two prediction paradigms yielded six distinct scenarios.

For each of the three scenarios, we trained models for both prognostic dichotomization paradigms I and II with favorable (mRS ≤ 2) and poor outcome (mRS 5 or death), respectively. This yielded six different scenarios in total (see Figure 1).

Multicollinearity was estimated using the variance inflation factor (VIF) (5).

### Applied Algorithms

We utilized all seven available ML algorithms from the framework to provide a comprehensive coverage of various ML methods. The more traditional techniques were represented by three algorithms: A generalized linear model (GLM), which for dichotomous outcomes is equivalent to a plain logistic regression, and two regularized variants, a Lasso algorithm with L1 regularization and an ElasticNet with L1 and L2 regularization.

Further, ML algorithms included tree boosting (Catboost implementation), a Support Vector Machine Classifier (SVMC), Naive Bayes (NB), and a Multilayer Perceptron (MLP).

### Model Training and Validation

The data comprising the given clinical parameters and outcomes were randomly split into training and test sets in a corresponding 4:1 ratio. Due to slight imbalance with respect to the outcome measures (127/210 patients with favorable outcome in paradigm I and 49/210 patients with poor outcome in paradigm II), random sub-sampling of the majority class was employed for the training sets. Test sets were stratified to follow the original imbalanced ratio to represent real distribution of our patient outcomes in model testing. In total, there were only 11 missing values in the data set. Missing values were imputed by mean/mode imputation. Non-categorical features—both in training and test sets—were standardized to zero-mean and unit variance based on training set statistics. Models were trained and best parameters were selected using 10-fold cross validation over an extensive grid of hyperparameters for each model. Parameter ranges were initially taken from the public repository referenced under the heading “ML framework”, and then refined taking run times of experiments into consideration. The used ranges are included in Supplementary Material. The whole process was repeated 200 times (shuffles) to account for dependence on the random procedure of train and test splits.

### Performance Assessment

For performance measures, we report results as the median over the test sets of the 200 shuffles. Model performance was primarily assessed by area-under-the-curve (AUC) measure resulting from receiver-operating-characteristic (ROC) analysis. Accuracy, balanced class accuracy, precision, recall, f1 score, negative predictive value, and specificity measures for each model are included in the Supplementary Material. Statistical significance of the difference between model performances on the respective variable sets was determined by the Wilcoxon signed-rank test at a confidence level of 5%.

### Explainability Assessment

We used SHapley Additive exPlanations (SHAP) scores to rate the importance of included clinical features for all seven models. More detailed explanation of the technique can be found in (14). The absolute values of importance scores on test sets were scaled to unit norm to yield comparable measures for all models, and then rescaled to the range of [0, 1] so that importance scores for a certain model sum to 1. Finally, mean and standard deviation across the 200 shuffles for stability and robustness were calculated and reported as the final importance rating.

## Results

### Patients

In total, 236 patients met the inclusion criteria, and 26 patients were excluded due to motion artifacts on MRI images or because no accurate AIF could be obtained, resulting in the final number of 210 patients. Median mismatch ratio was 4.6 (2.3–8.4). In 154 patients (73%), successful reperfusion (TICI 3 or 2b) could be achieved. Median time to TICI was 492 min. In prediction paradigm I, 83/210 patients (39%) had a favorable outcome (mRS 0–2). In prediction paradigm II, 49/210 patients (23%) had a poor outcome (mRS 5–6).

In this study, 168/210 patients (80%) had a target MRI mismatch [according to the EXTEND-IA study criteria (6)]; 5/210 patients (2%) had no ischemic core and 11/210 patients (5%) without target mismatch had a small ischemic core of 10 ml or less.

In the multicollinearity analysis, VIF values were below 5 for all scenarios using the predictive variable sets A and A+B. For the two scenarios I A+B+C and II A+B+C, time from stroke onset to final TICI and time from onset to MRI raised to values ~9.9, indicating stronger multicollinearity for these features. We did not recognize a harmful level of multicollinearity in any of the variable sets; thus, no features were eliminated.

### Prediction Models

The specific AUC results for the total of six prediction scenarios, each examined with seven algorithms are presented in Table 2 for paradigm I with favorable outcome and in Table 3 for paradigm II with poor outcome, respectively. The results for the additional performance measures are given in Supplementary Material.

**Table 2:** Models for favorable outcome (paradigm I).

**Scenario**	**GLM**	**Lasso**	**ElasticNet**	**Catboost**	**MLP**	**SVMC**	**Naive bayes**
I A	0.65	0.65	0.6	**0.67**	**0.67**	0.6	0.65
I A+B	0.62*	0.64*	0.6	0.64*	0.64*	0.57	0.63*
I A+B+C	0.71*+	0.71*+	0.68*+	0.73*+	0.7*+	0.67*+	0.69*+

The prediction variable sets (Table 1) were used to predict a favorable outcome (mRS dichotomized as 0–2 vs. 3–6). The addition of thrombectomy-associated variables (set C) leads to a noticeable improvement of all machine learning models. Highest AUC results for each variable set are marked in bold. Confidence intervals for all models are included in the Supplementary Material. Statistically significant difference in model performance between variable sets are marked with * and + to signal difference from A and from A+B, respectively. Significance was determined by a value of p lower than 0.05, resulting from the Wilcoxon signed-rank test.

**Table 3:** Models for poor outcome (paradigm II).

**Scenario**	**GLM**	**Lasso**	**ElasticNet**	**Catboost**	**MLP**	**SVMC**	**Naive bayes**
**II A**	0.67	0.7	0.64	0.7	**0.71**	0.59	0.69
**II A+B**	0.65*	**0.7**	0.62*	**0.7**	0.69	0.57	0.65*
**II A+B+C**	0.68+	0.71	0.65+	0.73*+	0.7	0.65*+	0.66*

The prediction variable sets (Table 1) were used to predict poor outcome (mRS dichotomized as 5 and 6 vs. 0–4). In contrast to the favorable outcome paradigm, the addition of thrombectomy-associated variables (set C) did not lead to relevant improvements in the performance of machine learning models. Only the Catboost model profited slightly in a clinically relevant AUC range. Highest AUC results for each variable set are marked in bold. Confidence intervals for all models are included in the Supplementary Material. Statistically significant difference in model performance between variable sets are marked with * and + to signal difference from A and from A+B, respectively. Significance was determined by a value of p lower than 0.05, resulting from the Wilcoxon signed-rank test.

For the first scenario with baseline clinical variables only (I A and II A), prediction was slightly better for poor outcome (II A) than for favorable outcome (I A). The smallest difference in AUC between I A and II A was 0.02 for GLM and the largest 0.05 for Lasso logistic regression. Only SVMC showed comparable results.

Adding MRI-derived parameters (scenario I A+B and II A+B) did not change the prediction performance for both paradigms. This was consistent for all algorithms in both scenarios I A+B and II A+B.

Finally, adding thrombectomy-associated parameters—extent and speed of recanalization—(I A+B+C and II A+B+C) improved the prediction performance noticeably across many algorithms for the favorable outcome paradigm (I A+B+C). Prediction for the poor outcome paradigm with all variables (II A+B+C) remained approximately stable; only the Catboost and SVMC algorithm showed a slight improvement (AUC increase of 0.03, 0.08, respectively, compared to II A).

To summarize, prediction for the poor outcome paradigm II remained comparable on a relatively high level across all three prediction scenarios (II A, II A+B, II A+B+C). Contrariwise, prediction for the favorable outcome paradigm I improved noticeably when thrombectomy-associated parameters were added (I A+B+C). The final performance for the last scenario with all predictive variables included (I A+B+C and II A+B+C) was comparable for both the favorable and poor paradigms.

### Feature Importance Ranking

**Figure 2:**
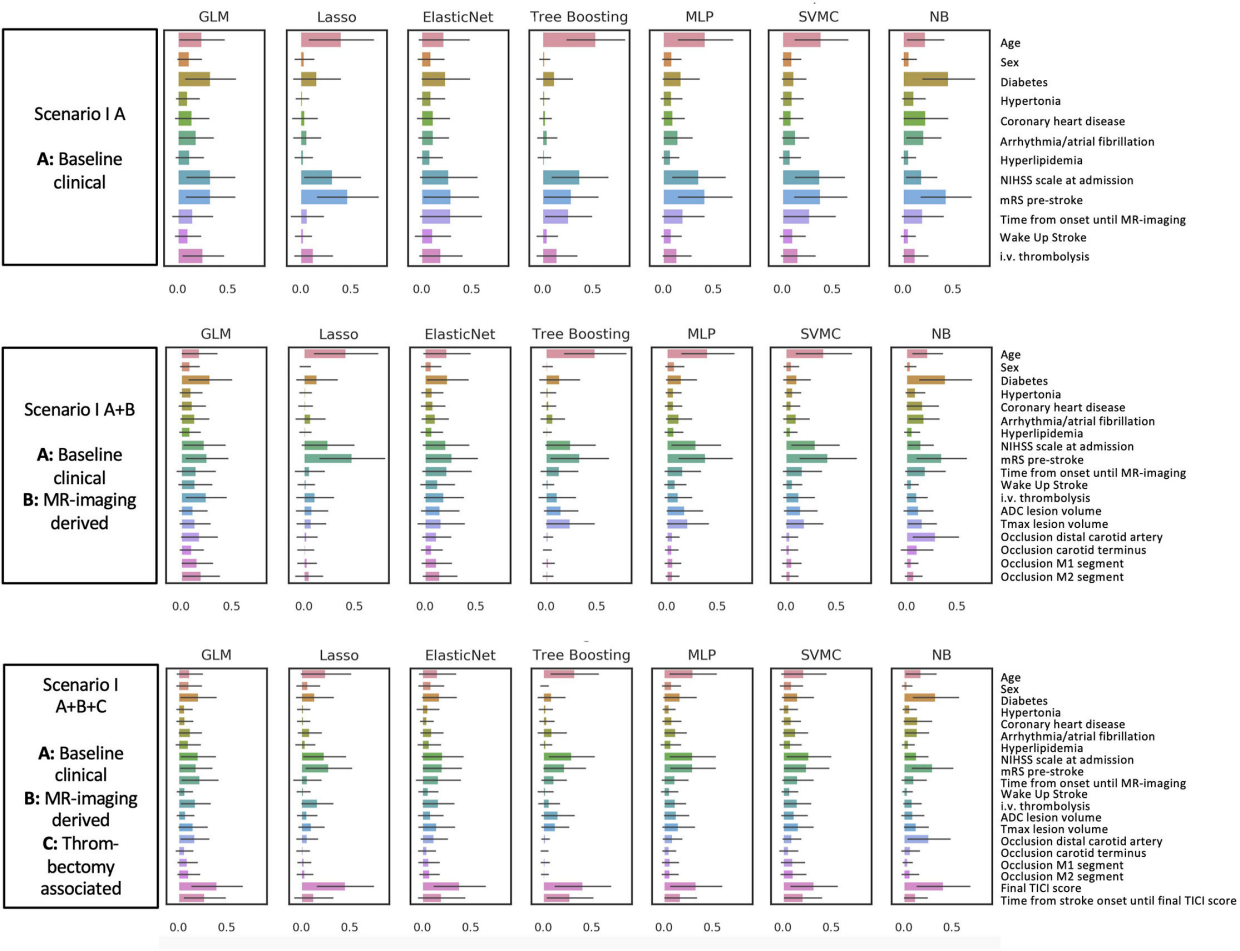
Favorable outcome mRS 0–2 (Prediction paradigm I). Feature importance for outcome paradigm I (favorable outcome) and II (poor outcome). The figures give an overview of the importance of the predictive variables included in the six different scenarios. Feature importance is given as a scaled SHAP score from 0 to 1. Values closer to 1 indicate higher importance for prediction.

**Figure 3:**
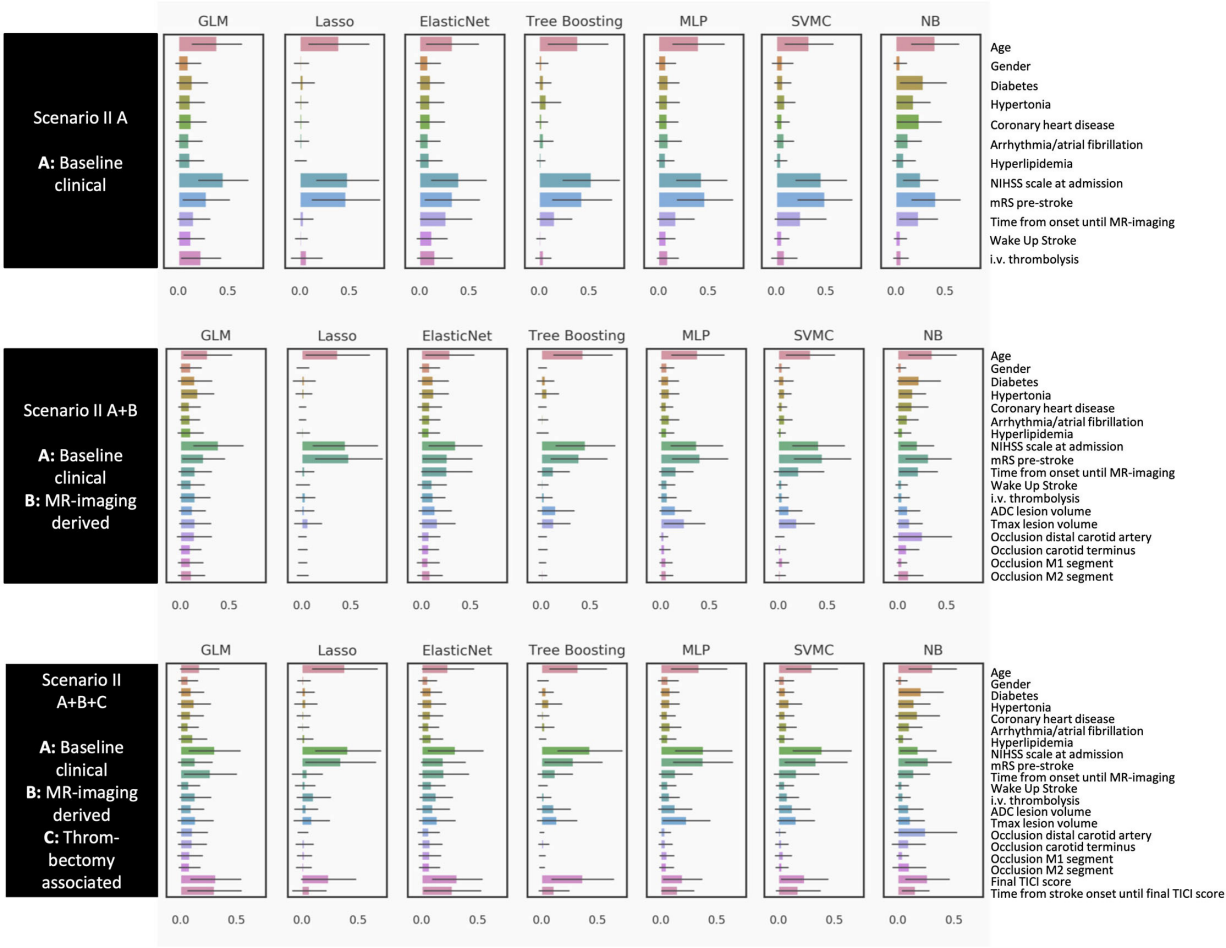
Poor outcome mRS 5 and 6 (Prediction paradigm II). Feature importance for outcome paradigm I (favorable outcome) and II (poor outcome). The figures give an overview of the importance of the predictive variables included in the six different scenarios. Feature importance is given as a scaled SHAP score from 0 to 1. Values closer to 1 indicate higher importance for prediction.

Feature importance values for each scenario and each algorithm are displayed in Figures 2, 3.

For the favorable outcome paradigm I (Figure 2), the most relevant variables across all algorithms for the first scenario with the baseline clinical variables only (I A) were age, pre-stroke mRS, National Institutes of Health Stroke Scale (NIHSS) at admission, and time from stroke onset to MRI. Adding MRI-derived mismatch parameters and the site of occlusion (I A+B), the Tmax volume for hypoperfused tissue was of higher relevance, while the ADC volume was of moderate importance. However, this was only visible in some models, among them the tree boosting model (the best performing model in the favorable outcome paradigm I). For models with all variables combined (I A+B+C), the mTICI score became the most dominant parameter in all models. Also, time from stroke onset to final TICI score was assigned high importance by the majority of models.

For the poor outcome prediction paradigm (Figure 3), we found a similar pattern. Age, premorbid mRS, and the baseline NIHSS were the most relevant features in the model with baseline variables only (II A). However, i.v. thrombolysis and risk factors such as diabetes played a smaller role compared to the favorable outcome paradigm (I A). For the models with additional MR-mismatch parameters (II A+B), both ADC and Tmax volume were of less importance than in the favorable outcome paradigm (I A+B). In the third scenario with all variables included (II A+B+C), the baseline variables from the first scenario (II A) remained important. Additionally, the mTICI score was relevant in most models, however not as relevant as compared to the favorable outcome prediction (I A+B+C).

To summarize, for the prediction of either good or poor outcome, age, premorbid mRS, and baseline NIHSS were important, with mTICI score as an additional relevant feature from the third scenario. ADC and Tmax volume were more important for the favorable than for the poor outcome paradigm. Information about i.v. thrombolysis was only important for the favorable outcome paradigm.

## Discussion

In this study, we examined ML-based outcome prediction models for patients with stroke who underwent MT. We compared prediction of poor outcome (mRS 5 or 6 vs. 0–4) and favorable outcome (mRS 0–2 vs. 3–6) measured at 3 months post stroke. These prediction paradigms have direct implications for clinical decision-making by predicting an outcome of no or only slight disability on the one hand and severe disability or death on the other. In particular, the definition of favorable outcome is generally accepted and was applied in large prospective studies. We chose different combinations of prognostic variables that were deliberately limited to those most commonly used in stroke practice and literature (1, 6, 7) and most accessible in clinical decision-making, especially under time constraints as encountered in clinical practice. We found considerable differences between the two outcome paradigms.

Our main finding suggests that prediction of poor outcome may possibly be based on clinical baseline variables only and set a rather high benchmark in the first prediction scenario. The predictive performance did not improve by adding target MR-mismatch and recanalization-related parameters. In contrast, prediction of favorable outcome did improve significantly by adding speed and extent of recanalization compared to using baseline clinical variables only.

In contrast to previous studies, the main strength of the presented work is the direct comparison of two different outcome prediction paradigms. The choice of how to dichotomize mRS for outcome prediction has clinical relevance: The standard dichotomization with 0–2 vs. 3–6 as used in the large randomized clinical trials puts an emphasis on patients with favorable outcomes. Dichotomizing mRS 0–4 vs. 5–6 focuses on patients with a very high degree of dependency or mortality after MT.

Our exploratory analysis revealed that patients with expected poor outcome could already be captured by clinical baseline variables before thrombectomy. The prediction scenario with baseline clinical variables only was already close to the final performance when mismatch and recanalization information was added. In those patients, withholding MT based on the clinical baseline variables could be the consequence; however, these implications should be verified in larger, prospective studies (8) and within new clinical data sets.

This was in contrast to the prediction of favorable outcome, where the prediction with baseline clinical variables only was lower but could be considerably improved by adding information about the speed and extent of reperfusion. However, because of the retrospective and thus exploratory nature of our study, our results should be interpreted with caution with regards to clinical treatment decisions. Nonetheless, they warrant further exploration in prospective studies to confirm our findings. Within such prospective data, the proposed paradigms could then be used to estimate individualized chances for either poor or favorable outcome before and after therapy. A similar approach using two models for an individualized prognosis of the same outcome variable is proposed by Debs et al. (15).

This performance pattern was accompanied by complimentary information from the feature importance analysis: For poor outcome, age, stroke severity (NIHSS) and degree of disability before stroke (premorbid mRS before stroke), and the time from stroke onset to imaging were the most important baseline variables. For favorable outcome, the most important predictive variables were also age, NIHSS at onset, and the mRS before stroke, but additionally, the speed and extent of recanalization (mTICI score and the time from stroke onset to the final mTICI score) were paramount. NIHSS was a more important predictor for final outcome than the ischemic lesion volume before therapy. This could be due to lesion location in eloquent brain regions where a smaller infarct causes comparably more severe clinical symptoms. Also, the final ischemic lesion volume after therapy might improve prediction but was not available to be included in our models.

Across all models, the maximum predictive value was an AUC of 0.73 for both favorable and poor outcome with regards to model performance and feature rankings. Our findings are comparable to previous studies applying ML algorithms: Hammam et al. (16) found a similar prediction for favorable outcome for patients with MT which did not considerably improve by adding MR-mismatch and other imaging-derived parameters. Other studies applied ML for outcome prediction with baseline CT imaging: Brugnara et al. (17) found an AUC of 0.85 for the prediction of favorable outcome only after adding information about infarct size after thrombectomy. Ramos et al. (18) did evaluate prediction for poor outcome with a multitude of clinical baseline parameters and CT-derived imaging features in a much larger cohort. Not including mismatch variables, their highest AUC was 0.81. Van Os et al. similarly showed a considerable improvement for prediction by adding treatment-associated variables in a study including CT imaging (19).

Interestingly, prediction for either poor or favorable outcome did not improve by including MR-mismatch variables. These results need to be interpreted together with the characteristics of the cohort: Most patients included had a target mismatch as defined in the inclusion criteria for the EXTEND-IA study (6) with an infarct core of <70 ml on ADC maps and a comparably larger volume of hypoperfused tissue on Tmax maps with a mismatch volume ratio of 1.2 or higher. While the treatment effect of MT is maintained even in patients with larger infarct cores (20), individual patients with a target mismatch still have poor outcome. Therefore, it is intriguing that poor outcome prediction in our study was possible based on clinical baseline variables only: For those patients, the potential predictive value of MR mismatch variables could be already encoded in the clinical baseline information. A similar conclusion can be drawn for patients with favorable outcome: improved prediction was much more dependent on speed and the extent of recanalization than on MR mismatch. However, this does not preclude the possibility that a target mismatch is still a valid selection criterion for patients undergoing MT. Our sample does not allow a conclusion about the potential predictive value of patients without MR mismatch. Prediction models could be improved by including patients who underwent MT without a target mismatch profile and larger infarct cores (21).

Despite these findings, the overall performance of the ML models tested in our study could be improved. Considering the potential power of ML algorithms to extract patterns, our findings suggest that important variables for outcome prediction might not be included in today's clinical decision-making. It is conceivable that there are so far unknown or undetectable variables. This warrants further studies including more and new prediction variables and biomarkers as well as direct integration of multimodal imaging and clinical information.

A deep learning model including raw imaging data and not derived variables might extract further, previously unknown predictive information. For example, these models might be able to account for inherent errors in the definition of infarct core (22) or individual susceptibility of brain tissue (21).

Finally, our results show that the predictive value can differ significantly between two different dichotomization paradigms or different “cutoffs” (mRS 0–2 vs. 3–6 and mRS 5/6 vs. 0–4). Unnecessary dichotomization of the mRS can be suboptimal (23). Researchers and clinicians should be aware that there are relevant differences between dichotomization paradigms. Defining more accurate outcome or premorbidity scores might improve future prediction models.

Our study has some limitations. It is based on a relatively small and retrospective patient cohort. Thus, our results must be understood as an exploratory analysis for future research.

Our data reach back to 2009. This might have influenced outcome due to improvements in thrombectomy technique and accelerated workflows. However, there was no significant outcome difference between patients treated 2009–2013 vs. 2014–2018.

## Conclusion

Our results suggest that a prediction of poor outcome (mRS of 5 or 6) after MT can be based on clinical baseline variables only. Speed and extent of thrombectomy did not seem to influence poor outcome but were important for favorable outcome (mRS 0-2). The predictive value of a target MR mismatch with smaller infarct core and larger penumbra was not relevant and could be already captured by clinical baseline variables. However, our sample does not allow a conclusion about the predictive value in patients without target MR mismatch.

## Data Availability Statement

The raw data supporting the conclusions of this article will be made available by the authors upon reasonable request.

## Ethics Statement

The studies involving human participants were reviewed and approved by Ethics Committee of Heidelberg University. Written informed consent for participation was not required for this study in accordance with the national legislation and the institutional requirements.

## Author Contributions

MMu, AP, CW, MMö, PR, SN, and MB acquired the data and organized the database. VM, AH, EZ, and DF performed and developed the machine learning and statistical analysis. MMu wrote the first draft of the manuscript. VM, AH, and DF wrote sections of the manuscript. All authors contributed to the conception, design of the study, manuscript revision, read, and approved the submitted version.

## Conflict of Interest

VM reported receiving personal fees from ai4medicine outside the submitted work. AH reported receiving personal fees from ai4medicine outside the submitted work. DF reported receiving grants from the European Commission Horizon2020 PRECISE4Q No. 777107, reported receiving personal fees from and holding an equity interest in ai4medicine outside the submitted work. There is no connection, commercial exploitation, transfer, or association between the projects of ai4medicine and the results presented in this work. SN received unrelated fees for consultancy from Brainomix and Boehringer Ingelheim, payment for lectures including service on speakers' bureaus from Pfizer, Medtronic, and Bayer AG. MB received unrelated grants from Siemens, grants and personal fees from Novartis, grants from Stryker, grants from DFG, personal fees from Merck, personal fees from Bayer, personal fees from Teva, grants and personal fees from Guerbet, personal fees from Boehringer, personal fees from Vascular Dynamics, personal fees from Grifols, and grants from the European Union, all outside the submitted work. MMö received unrelated Board Membership from Codman; consultancy from Medtronic, MicroVention, and Stryker; payment for lectures including service on speakers bureaus' from Medtronic, MicroVention, and Stryker. PR received unrelated grants for consultancy from Boehringer and lecture fees from Bayer, Boehringer Ingelheim, BMS, Daichii Sankyo, and Pfizer. The remaining authors declare that the research was conducted in the absence of any commercial or financial relationships that could be construed as a potential conflict of interest.

## Publisher's Note

All claims expressed in this article are solely those of the authors and do not necessarily represent those of their affiliated organizations, or those of the publisher, the editors and the reviewers. Any product that may be evaluated in this article, or claim that may be made by its manufacturer, is not guaranteed or endorsed by the publisher.

## Abbreviations

MT: mechanical thrombectomy
AIS: acute ischemic stroke
LVO: large vessel occlusion
mRS: modified Rankin Scale
DWI: diffusion weighted imaging
MRI: magnetic resonance imaging
Tmax: time-to-maximum
mTICI: modified treatment in cerebral ischemia
NIHSS: National Institutes of Health Stroke Scale
GLM: generalized linear model
SVMC: Support Vector Machine Classifier
NB: Naive Bayes
MLP: Multilayer Perceptron
AUC: area-under-the-curve
ROC: receiver-operating-characteristic
SHAP: Shapley Additive Explanations
VIF: variance inflation factor.
