# Supplementary material for: Comparing Poor and Favorable Outcome Prediction With Machine Learning After Mechanical Thrombectomy in Acute Ischemic Stroke
Source: Front Neurol. 2022 May 27;13:737667. doi: 10.3389/fneur.2022.737667 (PMC9184444; doi:10.3389/fneur.2022.737667)
Supplement: Supplementary file 1 [file Data_Sheet_1.PDF]

SUPPLEMENTAL MATERIAL

Imaging protocol:

Diffusion weighted imaging was performed using a single-shot spin echo echo-planar (EPI) sequence with spectral fat saturation and repetition and echo times (TR/TE) =5300 ms/90 ms, flip angles of 90°/180° and a slice thickness (ST) of 5 mm. Diffusion sensitizing gradients were applied sequentially with b=0 and b=1200 s/mm^2. 2D-T2 TSE-weighted and 2D-FLAIR-weighted images were acquired with TR=5000 ms/8500 ms, TE=85 ms/133 ms, ST=5 mm and a slice gap of 0.5 mm.

DSC-PWI was performed with a T2*-weighted gradient-echo EPI sequence with fat supression (TR/TE = 2220 ms/36 ms, flip angle 90°, field of view: 240×240 mm^2, image matrix: 128×128 mm, 25-27 slices with ST of 5 mm.) and was started simultaneously with bolus injection of a standard dose (0.1 mmol/kg) of an intravenous gadolinium-based contrast agent. In total, 50–75 dynamic measurements were performed (including at least eight prebolus measurements). Bolus and prebolus were injected with a pneumatically driven injection pump at an injection rate of 5 ml/s.

Supplemental results

Scenario I A

Model		AUC	accuracy	average class accuracy	f1	precision PPV	NPV	recall sensitivity	specificity	brier score loss
GLM	median	0,65	0,60	0,58	0,57	0,50	0,71	0,65	0,56	0,24
GLM	IQR	0,11	0,10	0,11	0,11	0,09	0,10	0,12	0,16	0,04
Lasso	median	0,65	0,60	0,60	0,57	0,50	0,70	0,65	0,56	0,24
Lasso	IQR	0,11	0,10	0,09	0,10	0,09	0,11	0,18	0,16	0,03
ElasticNet	median	0,60	0,55	0,53	0,54	0,46	0,67	0,65	0,52	0,33
ElasticNet	IQR	0,15	0,12	0,13	0,14	0,10	0,11	0,29	0,20	0,16
Catboost	median	0,67	0,64	0,62	0,59	0,54	0,72	0,65	0,64	0,23
Catboost	IQR	0,11	0,10	0,09	0,09	0,10	0,08	0,18	0,13	0,02
MLP	median	0,67	0,62	0,58	0,58	0,52	0,71	0,65	0,56	0,25
MLP	IQR	0,11	0,10	0,11	0,10	0,09	0,11	0,24	0,20	0,02
SVMC	median	0,60	0,57	0,53	0,56	0,46	0,68	0,65	0,52	0,25
SVMC	IQR	0,24	0,10	0,16	0,12	0,10	0,13	0,24	0,25	0,02
NB	median	0,65	0,60	0,59	0,57	0,50	0,71	0,65	0,56	0,29
NB	IQR	0,10	0,10	0,10	0,10	0,08	0,11	0,18	0,13	0,07

Scenario I A+B

Model		AUC	accuracy	average class accuracy	f1	precision PPV	NPV	recall sensitivity	specificity	brier score loss
GLM	median	0,62	0,57	0,57	0,53	0,48	0,67	0,59	0,56	0,26
GLM	IQR	0,10	0,07	0,11	0,12	0,09	0,10	0,12	0,12	0,04
Lasso	median	0,64	0,60	0,58	0,55	0,50	0,69	0,65	0,56	0,24
Lasso	IQR	0,10	0,12	0,10	0,11	0,09	0,10	0,18	0,16	0,02
ElasticNet	median	0,60	0,55	0,53	0,54	0,46	0,67	0,59	0,52	0,35
ElasticNet	IQR	0,12	0,12	0,14	0,12	0,11	0,11	0,19	0,20	0,13
Catboost	median	0,64	0,62	0,60	0,57	0,52	0,70	0,65	0,60	0,24
Catboost	IQR	0,11	0,10	0,10	0,11	0,10	0,10	0,18	0,16	0,02
MLP	median	0,64	0,60	0,55	0,55	0,50	0,68	0,62	0,60	0,25
MLP	IQR	0,10	0,10	0,14	0,11	0,10	0,09	0,24	0,24	0
SVMC	median	0,57	0,57	0,57	0,55	0,48	0,68	0,65	0,52	0,25
SVMC	IQR	0,19	0,10	0,14	0,09	0,10	0,10	0,18	0,20	0,02
NB	median	0,63	0,60	0,57	0,54	0,50	0,68	0,59	0,60	0,3
NB	IQR	0,11	0,10	0,11	0,13	0,11	0,09	0,18	0,16	0,07

Scenario I A+B+C

Model		AUC	accuracy	average class accuracy	f1	precision PPV	NPV	recall sensitivity	specificity	brier score loss
GLM	median	0,71	0,64	0,65	0,62	0,56	0,75	0,71	0,64	0,23
GLM	IQR	0,10	0,07	0,10	0,11	0,08	0,10	0,18	0,12	0,05
Lasso	median	0,71	0,64	0,63	0,59	0,55	0,73	0,65	0,64	0,22
Lasso	IQR	0,10	0,10	0,10	0,13	0,09	0,12	0,18	0,12	0,04
ElasticNet	median	0,68	0,62	0,60	0,58	0,52	0,71	0,65	0,60	0,29
ElasticNet	IQR	0,10	0,10	0,10	0,11	0,10	0,10	0,24	0,17	0,14
Catboost	median	0,73	0,67	0,65	0,63	0,57	0,75	0,71	0,64	0,21
Catboost	IQR	0,10	0,10	0,12	0,12	0,12	0,11	0,18	0,16	0,03
MLP	median	0,70	0,64	0,62	0,60	0,55	0,74	0,65	0,62	0,24
MLP	IQR	0,12	0,10	0,13	0,14	0,11	0,13	0,24	0,16	0,04
SVMC	median	0,67	0,62	0,61	0,59	0,52	0,72	0,71	0,56	0,23
SVMC	IQR	0,10	0,10	0,10	0,10	0,10	0,11	0,18	0,13	0,03
NB	median	0,69	0,64	0,62	0,58	0,56	0,71	0,59	0,68	0,27
NB	IQR	0,10	0,10	0,10	0,11	0,12	0,09	0,18	0,16	0,07

Scenario II A

Model		AUC	accuracy	average class accuracy	f1	precision PPV	NPV	recall sensitivity	specificity	brier score loss
GLM	median	0,67	0,60	0,60	0,43	0,32	0,84	0,60	0,62	0,25
GLM	IQR	0,13	0,10	0,11	0,11	0,10	0,07	0,20	0,13	0,05
Lasso	median	0,70	0,60	0,59	0,44	0,33	0,84	0,70	0,56	0,24
Lasso	IQR	0,12	0,10	0,12	0,11	0,09	0,09	0,20	0,16	0,03
ElasticNet	median	0,64	0,60	0,58	0,42	0,31	0,83	0,60	0,59	0,36
ElasticNet	IQR	0,12	0,10	0,13	0,12	0,09	0,08	0,20	0,19	0,16
Catboost	median	0,70	0,64	0,62	0,44	0,36	0,84	0,60	0,66	0,22
Catboost	IQR	0,12	0,07	0,11	0,12	0,10	0,07	0,20	0,09	0,03
MLP	median	0,71	0,57	0,58	0,44	0,32	0,87	0,80	0,52	0,25
MLP	IQR	0,12	0,19	0,14	0,10	0,08	0,09	0,30	0,28	0
SVMC	median	0,59	0,73	0,56	0,40	0,38	0,82	0,50	0,78	0,24
SVMC	IQR	0,35	0,12	0,22	0,19	0,20	0,07	0,30	0,25	0,04
NB	median	0,69	0,64	0,61	0,44	0,36	0,84	0,60	0,66	0,26
NB	IQR	0,11	0,10	0,12	0,12	0,10	0,07	0,20	0,16	0,08

Scenario II A+B

Model		AUC	accuracy	average class accuracy	f1	precision PPV	NPV	recall sensitivity	specificity	brier score loss
GLM	median	0,65	0,60	0,58	0,41	0,32	0,83	0,60	0,59	0,27
GLM	IQR	0,13	0,10	0,11	0,11	0,09	0,07	0,20	0,16	0,07
Lasso	median	0,70	0,60	0,60	0,44	0,33	0,85	0,70	0,56	0,24
Lasso	IQR	0,11	0,12	0,10	0,11	0,09	0,07	0,20	0,16	0,03
ElasticNet	median	0,62	0,57	0,55	0,40	0,30	0,82	0,60	0,56	0,38
ElasticNet	IQR	0,13	0,12	0,10	0,10	0,08	0,07	0,20	0,22	0,19
Catboost	median	0,70	0,64	0,61	0,44	0,35	0,84	0,60	0,66	0,22
Catboost	IQR	0,11	0,10	0,12	0,12	0,11	0,08	0,20	0,16	0,03
MLP	median	0,69	0,55	0,55	0,43	0,31	0,86	0,80	0,50	0,25
MLP	IQR	0,12	0,14	0,16	0,09	0,08	0,11	0,30	0,25	0
SVMC	median	0,57	0,71	0,57	0,43	0,36	0,83	0,50	0,75	0,24
SVMC	IQR	0,33	0,12	0,22	0,19	0,17	0,08	0,30	0,25	0,03
NB	median	0,65	0,62	0,59	0,42	0,33	0,83	0,60	0,62	0,3
NB	IQR	0,13	0,10	0,15	0,13	0,10	0,09	0,30	0,16	0,09

Scenario III A+B+C

Model		AUC	accuracy	average class accuracy	f1	precision PPV	NPV	recall sensitivity	specificity	brier score loss
GLM	median	0,68	0,64	0,61	0,44	0,35	0,84	0,60	0,66	0,27
GLM	IQR	0,13	0,10	0,10	0,10	0,10	0,07	0,20	0,16	0,07
Lasso	median	0,71	0,62	0,64	0,47	0,35	0,87	0,70	0,62	0,23
Lasso	IQR	0,11	0,10	0,10	0,10	0,10	0,08	0,20	0,16	0,03
ElasticNet	median	0,65	0,62	0,59	0,44	0,33	0,85	0,70	0,59	0,33
ElasticNet	IQR	0,14	0,12	0,16	0,15	0,11	0,09	0,30	0,16	0,17
Catboost	median	0,73	0,67	0,65	0,48	0,39	0,86	0,70	0,69	0,21
Catboost	IQR	0,11	0,10	0,12	0,13	0,10	0,07	0,22	0,09	0,03
MLP	median	0,70	0,55	0,56	0,44	0,31	0,86	0,75	0,50	0,25
MLP	IQR	0,11	0,17	0,17	0,10	0,09	0,10	0,30	0,31	0
SVMC	median	0,65	0,67	0,59	0,44	0,37	0,83	0,60	0,69	0,24
SVMC	IQR	0,24	0,14	0,14	0,11	0,13	0,07	0,30	0,19	0,03
NB	median	0,66	0,64	0,60	0,43	0,33	0,83	0,60	0,66	0,3
NB	IQR	0,15	0,12	0,15	0,15	0,12	0,09	0,22	0,19	0,11

Variance inflation factor

Variable name	Variable Set I	Variable Set II	Variable Set III
Age	1.56	1.96	2.02
Sex	1.80	2.10	2.17
Diabetes	1.31	1.37	1.37
Hypertonia	2.98	4.06	4.09
Coronary heart disease	1.32	1.40	1.42
Arrhythmia/atrial fibrillation	1.79	2.02	2.04
Hyperlipidemia	1.59	1.67	1.71
NIHSS scale at admission	1.17	1.42	1.44
mRS pre-stroke	1.19	1.29	1.30
Time from stroke onset to MR-imaging	1.25	1.29	9.87
Wake Up Stroke	1.64	2.10	2.11
i.v.thrombolysis	2.23	2.86	2.86
ADC lesion volume	n/a	1.51	1.54
Tmax lesion volume	n/a	1.85	1.90
Occlusion distal carotid artery	n/a	1.32	1.35
Occlusion carotid terminus	n/a	2.20	2.24
Occlusion M1 segment middle cerebral artery	n/a	4.42	4.51
Occlusion M2 segment middle cerebral artery	n/a	2.19	2.27
Final mTICI score (TICI 3 and 2b)	n/a	n/a	1.50
Time from stroke onset to final mTICI score	n/a	n/a	9.91

Hyperparameter ranges

Lasso:

C: [0.1, 0.1456, 0.2121, 0.3089, 0.4498, 0.6551, 0.9541, 1.1514, 1.6768, 2.4421, 3.5565, 5.1795, 7.5431, 9.103, 13.2571, 19.307, 28.1177, 40.9492, 59.6362, 86.8511]

ElasticNet:

L1 ratio: [0.0, 0.05, 0.1, 0.15, 0.2, 0.25, 0.3, 0.35, 0.4, 0.45, 0.5, 0.55, 0.6, 0.65, 0.7, 0.75, 0.8, 0.85, 0.9, 0.95]

Alpha: [0.00001, 0.00004, 0.00016, 0.0006, 0.0025, 0.01]

Catboost:

L2 leaf reg: [3.,10.]
Depth: [2., 4.]

Leaf estimation iterations: [1.,2.]
Bagging temperature: [0.6,0.8,1.]
Learning rate: [0.1,0.3]

MLP:

Batch size: [16,32]
Num. neurons: [8, 16, 32, 64]
Learning rate: [0.001, 0.01]
Dropout rate: [0.1, 0.2]
L1 ratio: [0.0001,0.001]

SVMC:

C: [0.01, 0.1, 1, 10]
Kernel: ['linear', 'poly', 'rbf']
Degree: [2,3]
